# Separate and concurrent use of methamphetamine and fentanyl: behavioral and neurobiological consequences

**DOI:** 10.3389/fnbeh.2026.1966221

**Published:** 2026-10-20

**Authors:** John T. Madden, Benjamin J. Clark, Derek A. Hamilton, Nathan S. Pentkowski

**Affiliations:** Department of Psychology, University of New Mexico, Albuquerque, NM, United States

**Keywords:** addiction, opioids, psychostimulants, drug abuse, poly drug use

## Abstract

**Background:**

Methamphetamine, a potent psychostimulant, is one of the most used substances globally. When administered alone, methamphetamine has powerful rewarding and reinforcing properties and produces numerous health consequences. However, recent national trends reveal that methamphetamine is increasingly being used concurrently with fentanyl. Of particular concern is that many methamphetamine users are unwittingly exposed to fentanyl, due to the increasing rate at which psychostimulants are “laced” with the opioid. While the amplified health risks of the drug combination, such as potential overdoses, are well documented, their shared addictive potential is less clear.

**Methods:**

In this review, we assessed the current literature on the neurobiological mechanisms associated with concurrent methamphetamine and fentanyl use, as well as the behavioral effects of the combined drugs.

**Results:**

Agonism of μ opioid receptors is a critical system involved in modulating the behavioral effects of methamphetamine and fentanyl, including conditioned place preference, cue-reactivity and behavioral sensitization. The existing literature also suggests that methamphetamine and fentanyl may have synergistic interactions that enhance their addictive potential, effects that are likely mediated by μ opioid receptors.

**Conclusion:**

Concurrent methamphetamine-fentanyl use is a mounting crisis. Unfortunately, the neurobiological and behavioral consequences of this drug combination have not been adequately examined. While the existing literature on concurrent methamphetamine-opioid use indicates that the drugs have synergistic addictive potential, more research specifically examining methamphetamine-fentanyl combinations is needed.

## Highlights

- Recent national trends in mortality data reveal that methamphetamine is increasingly being used concurrently with fentanyl.
- Existing literature suggests that the μ opioid receptor system underlies the synergistic effects of concurrent methamphetamine-fentanyl use.
- Self-report studies indicate that methamphetamine-opioid users believe the drugs enhance each other’s effects, producing a more pleasurable “high.”
- Research suggests that methamphetamine-fentanyl cocktails may be more reinforcing and rewarding when administered together vs. alone.
- The neurobiological and behavioral consequences of concurrent methamphetamine-fentanyl use have been largely under examined.

## 1 The growing methamphetamine-fentanyl epidemic

Amphetamines are potent central nervous system stimulants and are among the most widely used substances globally (Chomchai and Chomchai, 2015). According to the United Nations Office on Drugs and Crime (2025), approximately 31 million people aged 15–64 used amphetamines in 2023, making them the third most widely used controlled drug category after cannabis and opioids. In the United States, an estimated 2.4 million people aged 12 or older reported past-year methamphetamine use in 2024 (Substance Abuse and Mental Health Services Administration [SAMHSA], 2025). Of all illegal amphetamine-type stimulants, none are more commonly misused, nor carry a higher risk of dependence than methamphetamine (Chomchai and Chomchai, 2015; Degenhardt et al., 2010; Vearrier et al., 2012). In the US, amphetamine use has risen steadily, with an average of 1.6 million Americans trying methamphetamine each year between 2015 to 2018, and more than half meeting the diagnostic criteria for methamphetamine use disorder (Degenhardt et al., 2010; Jones et al., 2020). Importantly, recent trends indicate that overdose rates involving methamphetamine are dramatically rising (Dai et al., 2022; Ellis et al., 2018; Kariisa et al., 2019), partially due to the presence of illicit opioids, primarily fentanyl (Dai et al., 2022; Ellis et al., 2018; Zibbell et al., 2022). Indeed, self-reported rates of concurrent methamphetamine-fentanyl use nearly doubled between 2011 and 2017 (Ellis et al., 2018), and between 2013 and 2018, positivity rates for fentanyl in methamphetamine-positive urine samples increased from 0.9% to 7.9% (LaRue et al., 2019). Unfortunately, methamphetamine users can unwittingly consume fentanyl, due to the increasing rates that psychostimulants are “laced” with the opioid (Ellis et al., 2018; Zibbell et al., 2022).

Concurrent methamphetamine-fentanyl use amplifies risks, such as worse mental health outcomes and potential overdoses (Shearer et al., 2020; Timko et al., 2018), but the addictive potential of this drug combination is less clear. Belonging to distinct drug classes, methamphetamine and fentanyl have unique pharmacodynamic properties and thus produce unique psychoactive experiences. However, when administered together, synergistic effects can occur with one drug enhancing the hedonic effects of the other and simultaneously ameliorating aversive effects (Ellis et al., 2018; Rhodes et al., 2007). Importantly, these synergistic effects could potentiate long-term drug-seeking and -taking behaviors. Unfortunately, the effects of methamphetamine-fentanyl combinations have yet to be adequately studied, nor have the neurobiological and behavioral mechanisms of these drug combinations been comprehensively reviewed, leaving a critical gap in the literature.

This review will address three specific aims. First, we will review the neurobiological and behavioral mechanisms of methamphetamine and fentanyl when administered in isolation to provide a basis of the drugs’ unique effects, to which concurrent methamphetamine and fentanyl use will be compared. Second, the neural mechanisms affected by concurrent methamphetamine-fentanyl use, and how these effects might translate into long-term neuroadaptations, will be described. Lastly, the behavioral changes induced by concurrent methamphetamine-fentanyl use that likely exacerbate the progression of addiction will be discussed (see Table 1 for a summary of the behavioral effects). Collectively, these aims will help characterize the addiction vulnerability posed by concurrent methamphetamine-fentanyl use and will provide a clear path for future studies examining this dangerous drug combination.

**Table 1:** Effects of mu opioid receptor agonists and antagonists on methamphetamine-induced behaviors.

Reference	Drug names and combinations	Design of study	Subjects	Route of administration	Drug dose	Results
Anggadiredja et al. (2004)	Methamphetamine, Naltrexone	Reinstatement of methamphetamine self-administration	Male Wistar rats; age not specified	Jugular catheter self-administration	0.02 mg over 6 s infusion methamphetamine 0.1 mL/100 g naltrexone	Naltrexone attenuated cue-induced reinstatement of drug seeking, but had no effect of methamphetamine-induced reinstatement
Chiu et al. (2005)	Methamphetamine, Naltrexone	Effect of naltrexone pretreatment on behavioral sensitization to methamphetamine	Male Swiss mice; age not specified	Intraperitoneal injection	10 or 20 mg/kg naltrexone 1.25 or 2.5 mg/kg methamphetamine	Expression of behavioral sensitization to methamphetamine attenuated by pretreatment with Naltrexone
Dawes et al. (2024)	Methamphetamine, Fentanyl	Effects of methamphetamine–fentanyl co-administration on self-administration behavior and nucleus accumbens dopamine function.	Male and female Long-Evans rats; age not specified.	Intravenous jugular catheter self-administration	1.25, 2.5, or 5 μg/kg/infusion fentanyl, alone or combined with 0.1 mg/kg/infusion methamphetamine.	Methamphetamine co-administration abolished fentanyl dose-dependent responding in both sexes and enhanced fentanyl intake in males. Combined exposure produced larger deficits in nucleus accumbens dopamine release and reuptake in males than either drug alone, with distinct dopamine responses observed in females.
Kaka et al. (2014)	Methamphetamine, Morphine, Naloxone	Behavioral effects of methamphetamine-morphine co-administration following Naloxone-induced withdrawal	Male Wistar Rats; age not specified	Intraperitoneal injection	Escalating daily doses. 5, 10, 20, 30, and 40 mg/kg morphine with 1, 2, 4, 6, and 8 mg/kg methamphetamine. Each daily dose split across two injections, divided by 12 h.	Frequency and incidence rate of jumping increased in rats co-administered methamphetamine and morphine, indicative of increased anxiety-like behavior.
Lan et al. (2009)	Methamphetamine, Morphine	Effects of methamphetamine-morphine co-administration on acquisition of CPP	Male Albino mice, 8–10 weeks old	Intraperitoneal injection	0.75 mg/kg methamphetamine, 5 mg/kg morphine, or both doses combined.	Co-administration of methamphetamine and morphine enhanced acquisition of CPP compared to either drug alone.
Paz-Ramos et al. (2024)	Fentanyl, Methamphetamine	Effects of methamphetamine-fentanyl co-administration on acquisition of CPP	Adult male Wistar rats	Subcutaneous injection	0.01 or 0.03 mg/kg methamphetamine with 0.3 μg/kg fentanyl	Ineffective doses of methamphetamine and fentanyl successfully produced acquisition and reinstatement of CPP when co-administered
Ranaldi and Wise (2000)	Heroin, Methamphetamine	Self-administration of methamphetamine and heroin “cocktails” under progressive ratio schedules	Male Long-Evans rats; age not specified	Jugular catheter self-administration	0.0625, 0.125, 0.25, 0.5,1.0 and 2.0 mg/kg/infusion methamphetamine, alone or with 12.5 μg/kg/infusion heroin	Rats reached higher break points at each dose of methamphetamine-heroin than methamphetamine administered alone
Ray et al. (2015)	Methamphetamine, Naltrexone	Effect of daily naltrexone on cue-induced craving and hedonic ratings of methamphetamine	Human methamphetamine users	Oral	25 mg naltrexone on day one, 50 mg on days two through four Two 15 mg IV infusions of methamphetamine, separated by 30 min	4-day oral Naltrexone administration attenuated cue-induced cravings and reduced subjective hedonic responses to methamphetamine
Roche et al. (2017)	Methamphetamine, Naltrexone	Effect of daily naltrexone on cue-induced craving and hedonic ratings of methamphetamine	Human methamphetamine users	Oral	25 mg naltrexone on day one, 50 mg on days two through four Two 15 mg IV infusions of methamphetamine, separated by 30 min	4-day oral Naltrexone administration attenuated cue-induced cravings and reduced subjective hedonic responses to methamphetamine
Seaman et al. (2022)	Fentanyl, Methamphetamine	Methamphetamine or fentanyl fixed ratio self-administration under choice procedure	Male Sprague–Dawley rats; age not specified	Femoral catheter self-administration	0.1 mg/kg/infusion methamphetamine 0.0032 mg/kg/infusion fentanyl	When the unit-dose of one drug is decreased, rats switch to self-administering the other, more “cost-effective,” drug option
Seaman and Collins (2021)	Fentanyl, Methamphetamine, Morphine	Effects of morphine withdrawal on progressive ratio self-administration of fentanyl and methamphetamine	Male Sprague–Dawley rats; age not specified	Femoral catheter self-administration	0.000032–0.01 mg/kg/infusion fentanyl 0.032 mg/kg/infusion methamphetamine	Morphine withdrawn rats displayed rightward shift in fentanyl dose–response. Dose–response of methamphetamine was unaffected.
Shen et al. (2010)	Methamphetamine	Effect or MOR knockout on development of methamphetamine behavioral-sensitization	Male MOR knockout mice; 8 to 12 weeks old	Intraperitoneal injection	0, 0.31, 0.62, 1.25, 2.5, or 10 mg/kg methamphetamine	Methamphetamine-induced behavioral sensitization was attenuated in MOR-knockout mice.
Tiihonen et al. (2012)	Amphetamines, Heroin, Naltrexone,	Effect of Prodetoxon naltrexone implant on polydrug use of amphetamines and heroin	Human amphetamine-heroin users	Subcutaneous implant	1,000 mg naltrexone; sustained release	Participants with implant decreased polydrug use and showed better treatment outcomes
Trivedi et al. (2021)	Bupropion and Extended-release Naltrexone	Impact of naltrexone on treatment outcomes as assessed by methamphetamine-negative urine samples	Human methamphetamine users	Intramuscular injections naltrexone Oral bupropion	380 mg naltrexone every 3 weeks 450 mg bupropion daily	Participants that received injectable naltrexone show better treatment outcomes than placebos
Trujillo et al. (2011)	Methamphetamine, Morphine	Effects of methamphetamine and morphine co-administration on ambulation, rearing, and fine movement	Adult male Sprague–Dawley rats	Subcutaneous “cocktail” injections	0.3 or 1.0 mg/kg methamphetamine administered with 1.0, 5.0 or 10.0 mg/kg morphine	Methamphetamine and morphine co-administration synergistically increased ambulation.
Wang et al. (2021)	Methamphetamine, Naltrexone	Effects of naltrexone on acquisition of methamphetamine CPP and behavioral sensitization	Adolescent male Kun-Ming mice; 6–8 weeks old	Intraperitoneal injection	10, 20, or 40 mg/kg naltrexone 1 mg/kg methamphetamine	Naltrexone administration reduced methamphetamine hyperlocomotion, suppressed behavioral sensitization, and attenuated expression of CPP.

## 2 Neurobiological and behavioral consequences of separate methamphetamine and fentanyl use

### 2.1 Neurobiological consequences of methamphetamine use

The pharmacokinetics of methamphetamine are well established (for review see Cruickshank and Dyer, 2009; NIDA, 2012). Briefly, while inhalation via smoking is the most popular route of administration, methamphetamine can also be administered orally, intranasally and intravenously (Cruickshank and Dyer, 2009; NIDA, 2012). Methamphetamine is highly lipophilic, permitting rapid brain entry, which increases its addictive properties (Schep et al., 2010). Methamphetamine also has a longer half-life than other psychostimulants (e.g., 12 h vs. 90 min for cocaine), giving it a longer duration of sustained action (Meredith et al., 2005).

The pharmacodynamic effects of methamphetamine are well characterized and primarily involve dysregulation of monoaminergic neurotransmission (Cruickshank and Dyer, 2009; Sulzer et al., 2005). Methamphetamine readily enters presynaptic monoaminergic neurons and acts as an agonist at trace amine-associated receptors 1 (TAAR1), an intracellular G protein-coupled receptor that regulates dopamine (DAT), norepinephrine (NET) and serotonin (SERT) transporters (Borowsky et al., 2001; Miller, 2011; Xie and Miller, 2007). Activation of TAAR1 increases cyclic adenosine monophosphate (cAMP) signaling and promotes protein kinase A (PKA)- and protein kinase C (PKC)-mediated phosphorylation of monoamine transporters, reducing neurotransmitter reuptake and promoting transporter-mediated efflux (Miller, 2011; Xie and Miller, 2007). Methamphetamine also disrupts vesicular monoamine transporter 2 (VMAT2) function, increasing cytosolic monoamine concentrations available for reverse transport into the synapse (Eiden and Weihe, 2011; Sulzer et al., 2005). Collectively, these mechanisms produce substantial elevations in extracellular dopamine, norepinephrine and serotonin.

The effects of methamphetamine on dopaminergic signaling throughout mesocorticolimbic circuitry is particularly relevant to addiction. Methamphetamine increases dopamine release within the nucleus accumbens (NAc), a region critically involved in reward, reinforcement and associative learning (Yorgason et al., 2020). Repeated exposure also produces persistent adaptations in dopaminergic signaling that contribute to behavioral sensitization and enhanced responsivity to drug-associated stimuli (Robinson and Becker, 1986; Robinson and Berridge, 2001). These effects are particularly relevant to concurrent opioid use because opioid receptor signaling can modulate mesolimbic dopamine transmission and several methamphetamine-induced behaviors. Indeed, pharmacological antagonism of MORs attenuates methamphetamine-induced conditioned place preference (CPP) and behavioral sensitization, suggesting that endogenous opioid signaling contributes to at least some of the behavioral consequences of methamphetamine exposure (Chiu et al., 2005; Shen et al., 2010; Tien and Ho, 2011; Wang et al., 2021). Importantly, these findings demonstrate opioidergic modulation of methamphetamine responses rather than an interaction between methamphetamine and fentanyl itself; however, they provide a mechanistic basis for investigating how a potent MOR agonist such as fentanyl can modify methamphetamine-induced reward and reinforcement.

### 2.2 Behavioral consequences of methamphetamine use

Rodent studies illustrate the addictive potential of methamphetamine, and how its use progresses across the stages of addiction. Rodents, regardless of age or sex, readily self-administer methamphetamine across a range of doses and dramatically escalate intake across repeated sessions, demonstrating the powerful reinforcing properties of the drug (Hasan et al., 2019; Kitamura et al., 2006; Reichel et al., 2012; Roth and Carroll, 2004). Methamphetamine rapidly induces CPP, even at low doses, effects that are long lasting, resistant to extinction, and are reinstated by drug-priming injections and stressors (Berry et al., 2012; Cullity et al., 2021; Taslimi et al., 2018; Zakharova et al., 2009). In the self-administration/extinction/reinstatement model, methamphetamine-seeking behavior is readily reinstated by drug-priming injections, exposure to drug-paired cues and stressors (Rogers et al., 2008; Rocha and Kalivas, 2010; Ruda-Kucerova et al., 2015; Shepard et al., 2004). Collectively, these findings indicate that methamphetamine is highly reinforcing/rewarding and produces incentive motivation to seek out the drug.

Chronic exposure to methamphetamine results in prominent symptoms of withdrawal. Specifically, rodents display little motivation for natural rewards and show decreased locomotor activity (Barr and Phillips, 1999; Orsini et al., 2001; Pulvirenti and Koob, 1993). In studies assessing intracranial self-stimulation, rodents undergoing early methamphetamine withdrawal exhibit higher reward thresholds and more depressive-like symptoms than drug-naïve controls (Jang et al., 2013). Lastly, both acute and chronic methamphetamine exposure produce long-term increases in anxiety-like behaviors during testing in the open field and elevated plus-maze (EPM) paradigms (Hayase et al., 2005; Hrubá et al., 2012; Jang et al., 2013; Loxton and Canales, 2017; Madden et al., 2020), which likely drives continued drug-seeking and -taking via negative reinforcement.

Methamphetamine readily produces behavioral sensitization. This phenomenon, also known as “reverse tolerance,” occurs following chronic methamphetamine exposure with the behavioral and physiological responses to a set dose of the drug increasing over time (Kalivas and Stewart, 1991; Robinson and Becker, 1986). Sensitization to the hyperlocomotive effects of methamphetamine is commonly seen in rodents (Chen et al., 2021; Hirabayasi et al., 1991; Robinson and Becker, 1986; Shuto et al., 2006), effects that are directly linked to structural and functional adaptations in the mesolimbic dopaminergic system and are believed to contribute to the development and maintenance of drug-seeking behaviors (Nestler, 2001; Nestler and Lüscher, 2019; Robinson and Becker, 1986; Robinson and Berridge, 2001).

Collectively, the existing literature highlights methamphetamine’s addictive potential. The psychostimulant directly impacts numerous neurotransmitter systems across reward-related circuits and induces long-term neuroadaptations that impair cognitive functioning and promote further drug-seeking. The behavioral effects of methamphetamine, such as enhanced locomotor activity, are sensitized over time, which may promote further drug-seeking. Furthermore, methamphetamine is highly reinforcing, resulting in high levels of drug-seeking (i.e., incentive motivation) and drug-taking behaviors that are readily reinstated after a period of abstinence/extinction by a variety of triggers including drug-priming injections, stressors and exposure to drug-associated cues.

### 2.3 Neurobiological consequences of fentanyl use

The pharmacokinetic profile of fentanyl has been extensively characterized (for review see Lötsch et al., 2013). The opioid can be administered intravenously, intranasally, orally or inhaled. Fentanyl has a longer half-life compared to other prominent opioids: 3 to 7 h for fentanyl, compared to 2 to 3 h for morphine and 3 to 4 min for heroin (Berkowitz, 1976; Milella et al., 2023; Ramos-Matos et al., 2023). Fentanyl is amongst the most lipid soluble opioids (Comer and Cahill, 2019; Lötsch et al., 2013), resulting in a faster onset of action than morphine, and unlike heroin, fentanyl does not need to be metabolized into psychoactive metabolites, factors that contribute to its potency and increased addiction potential (Comer and Cahill, 2019; van den Hoogen and Colpaert, 1987). Indeed, fentanyl is approximately 100 times more potent than morphine and 50 times more potent than heroin (van den Hoogen and Colpaert, 1987; Volpe et al., 2011).

Fentanyl exerts its primary pharmacodynamic effects through agonism of μ-opioid receptors (MORs), Gi/o protein-coupled receptors distributed throughout the central and peripheral nervous systems. Activation of MORs inhibits adenylyl cyclase activity, decreases intracellular cAMP concentrations, promotes potassium efflux and inhibits voltage-gated calcium channels, collectively reducing neuronal excitability and neurotransmitter release. Although fentanyl can interact with other opioid receptor subtypes, its high affinity and efficacy at MORs are principally responsible for its analgesic, rewarding and respiratory effects (Raynor et al., 1994; Volpe et al., 2011).

The VTA plays a prominent role in the progression of opioid addiction, exerting both excitatory and inhibitory control over key regions such as the NAc, prefrontal cortex and amygdala (Albanese and Minciacchi, 1983; Ford et al., 2006), regions linked to opioid reward processing, cue-induced recall and associative learning (Daglish et al., 2001; Luo et al., 2004; Milella et al., 2016; Volkow et al., 2004, 2006). Neurons within the VTA are typically grouped into three categories: primary, secondary and tertiary (Cameron et al., 1997). Primary and tertiary neurons are dopaminergic and populated with KORs that exert inhibitory control over VTA efferent projections (Margolis et al., 2003). Secondary neurons are GABAergic and are populated with MORs. Agonism of MORs inhibit GABAergic activity and disinhibit VTA dopaminergic efferent projections (Cameron et al., 1997; Ford et al., 2006; Gysling and Wang, 1983; Johnson and North, 1992). Rodent studies reveal that morphine infusions into the VTA promote CPP, while infusions of KOR agonists promote conditioned place aversion (Phillips and LePiane, 1980; Bals-Kubik et al., 1993). Compared to other prominent opioids, fentanyl exhibits weak binding affinity for KORs (Gharagozlou et al., 2006; Comer and Cahill, 2019), and unlike MOR activation, which is associated with euphoria and reward, KOR activation is generally associated with dysphoria and suppression of mesolimbic dopamine signaling. Thus, fentanyl’s comparatively lower binding affinity for KORs may modestly enhance its rewarding properties, however, its abuse liability is believed to arise primarily from high lipid solubility and high MOR efficacy rather than KOR selectivity (Raynor et al., 1994; Comer and Cahill, 2019).

Fentanyl use is associated with lasting alterations to mesolimbic dopaminergic signaling, particularly within the VTA. Acutely, fentanyl activates MORs on GABAergic neurons in the VTA disinhibiting dopaminergic neurons, an effect implicated in its positive reinforcing properties (Chaudun et al., 2024). Repeated opioid administration sensitizes this system, leading to elevated dopamine release in response to opioids or opioid-related cues (Cadoni and Di Chiara, 1999; Diana et al., 1999; Johnson and Glick, 1993; Zijlstra et al., 2008). Rodent studies reveal that chronic fentanyl exposure also reduces expression of G protein-coupled inwardly rectifying potassium channels, reducing inhibitory input onto dopamine neurons in the VTA, which can occur long after cessation of drug exposure. This lasting effect may result in enhanced excitability of dopaminergic neurons to subsequent drug use (Pachenari et al., 2025). Further, rats and mice self-administering fentanyl exhibit increased expression of genes associated with GABAergic signaling in the VTA, though their role in mediating opioid use has yet to be thoroughly investigated (Fox et al., 2024). Together, these findings indicate that fentanyl use enhances baseline dopaminergic activity, which likely promotes addiction vulnerability.

Although the present review emphasizes MOR-dependent modulation of mesolimbic dopamine signaling because of its potential relevance to methamphetamine–fentanyl interactions, opioid actions are not restricted to the VTA or to dopaminergic mechanisms. MORs are distributed across multiple brain regions and peripheral tissues, where their activation influences nociception, respiratory control, stress responses, and other physiological and behavioral processes (Fields and Margolis, 2015; Koob and Volkow, 2016; Valentino and Volkow, 2018). Accordingly, VTA–NAc dopamine signaling represents one potential point of interaction between methamphetamine and fentanyl rather than a comprehensive explanation of fentanyl’s effects or the consequences of their combined use.

### 2.4 Behavioral consequences of fentanyl use

Rodents quickly acquire CPP for fentanyl-paired contexts (Finlay et al., 1988) and readily self-administer fentanyl which escalates across subsequent sessions (Coffey et al., 2023; Townsend et al., 2019; Wade et al., 2015). Following extinction, drug seeking for these opioids is reinstated by classic triggers including drug-priming injections and cue-exposure (Malone et al., 2021). Prior history of drug exposure and affective state also play a prominent role in acquisition of opioid self-administration in healthy animals. Rodents withdrawn from morphine display increased responding for fentanyl compared to drug-naïve or drug-dependent animals (Seaman and Collins, 2021). Studies using ultrasonic vocalizations as indicators of affective state suggest that rats can exhibit a shift toward negative affect during ongoing fentanyl self-administration. Specifically, Coffey et al. (2023) observed a transition from predominantly 50-kHz vocalizations during the initial loading phase to 22-kHz vocalizations during the maintenance phase, despite continued fentanyl intake. This pattern is consistent with a potential shift toward negative reinforcement within a self-administration session, although vocalization patterns alone cannot establish the motivational mechanism underlying continued drug taking.

Humans and rodents display prominent symptoms of opioid withdrawal including elevated stress reactivity, agitation, impulsivity, and anxiety- and depressive-like symptoms including anhedonia (Bhalla et al., 2016; Harris and Gewirtz, 2005; Pergolizzi et al., 2020). However, the expression of fentanyl-associated anxiety-like behavior may depend on the timing of behavioral assessment relative to drug exposure and withdrawal. In adult male mice receiving daily fentanyl injections for 12–14 days, Fujii et al. (2019) reported reduced anxiety-like behaviors in the open-field and EPM; on test days fentanyl was administered after behavioral testing so mice were tested ~24 h following their last drug treatment. Following the cessation of treatment (14 days of withdrawal), mice that previously received a high-dose of fentanyl exhibited reduced time in the illuminated compartment during the first half of a light–dark test, consistent with increased anxiety-like behavior during extended withdrawal from chronic fentanyl exposure. However, the study did not detect corresponding group differences in the EPM during withdrawal, indicating that the observed effects varied across behavioral measures.

Sex and developmental stage may also influence responses to fentanyl. In a separate study in adolescent mice, Kestering-Ferreira et al. (2024) reported heightened locomotor activity in females compared to males following repeated fentanyl treatment, as well as more naloxone-precipitated withdrawal-induced jumping behaviors. These findings demonstrate sex-related differences under the conditions examined but do not establish that locomotor activity accounts for the differing anxiety-related outcomes reported by Fujii et al. (2019).

In summary, fentanyl is highly rewarding and reinforcing, promotes incentive salience, produces drug-seeking behaviors even after periods of abstinence, and induces withdrawal symptoms that may enhance long-term addiction vulnerability.

## 3 Defining concurrent methamphetamine-fentanyl use

An important consideration when evaluating the present literature on methamphetamine and fentanyl co-use is that studies differ substantially in how the exposure to the two drugs is modeled. In clinical populations, the term “concurrent use” is frequently used to describe individuals who report using both psychostimulants and opioids within a given period, although the precise dose, temporal relationship, route of administration and history of exposure to each drug are often difficult to establish (Earleywine and Newcomb, 1997). Consequently, while clinical studies of concurrent use provide important information regarding patterns and motivations for co-use, they cannot necessarily establish whether methamphetamine and fentanyl were pharmacologically active at the same time.

Preclinical studies provide greater experimental control over the temporal relationship between drug exposures but similarly employ paradigms that address distinct biological questions. For the purposes of the present review, concurrent use or exposure refers specifically to paradigms in which methamphetamine and fentanyl are administered together or are available within the same experimental session. These paradigms provide the most direct evidence regarding the behavioral and neurobiological consequences of methamphetamine-fentanyl co-use. Recent studies using concurrent-access self-administration and combined exposure in CPP paradigms therefore provide particularly important evidence regarding the reinforcing and rewarding effects of methamphetamine-fentanyl combinations (Dawes et al., 2024; Paz-Ramos et al., 2024; Seaman et al., 2022).

A second category includes sequential or drug-history paradigms, in which exposure to one drug precedes subsequent exposure or access to the other drug. These studies can determine whether previous psychostimulant or opioid exposure alters later drug reward, reinforcement, withdrawal or drug-seeking behavior, but they do not model concurrent pharmacological exposure. For example, studies examining the effects of opioid dependence or withdrawal on subsequent fentanyl or methamphetamine self-administration provide evidence that drug history modifies later behavioral responses, rather than evidence of an acute interaction between methamphetamine and fentanyl (Seaman and Collins, 2021).

Finally, mechanistic studies that use pharmacological or genetic manipulations of opioid or dopaminergic systems help to determine whether these systems contribute to the effects of either drug. Studies demonstrating that MOR antagonism or MOR deletion attenuates methamphetamine-induced CPP, sensitization or drug-seeking behavior provide evidence that opioid signaling contributes to methamphetamine-related behaviors (Chiu et al., 2005; Shen et al., 2010; Wang et al., 2021). However, these manipulations do not themselves model concurrent methamphetamine-fentanyl use. Instead, they identify candidate mechanisms through which fentanyl-mediated MOR activation could potentially modify the effects of methamphetamine.

These distinctions establish an important hierarchy for interpreting the available literature. Studies directly administering or providing access to methamphetamine and fentanyl together offer the strongest evidence regarding their interaction. Studies examining methamphetamine with other opioids, such as morphine or heroin, provide complementary but indirect evidence, whereas studies involving other psychostimulant-opioid combinations provide broader conceptual context. Mechanistic manipulations of opioid or dopaminergic systems further identify potential substrates of interaction but should not be interpreted as evidence of concurrent drug effects themselves. Because direct studies of methamphetamine-fentanyl combinations remain relatively limited, each level of evidence is considered throughout this review, with the source and limitations of extrapolation explicitly identified.

## 4 Neurobiological consequences of concurrent methamphetamine-fentanyl use

### 4.1 Neurotransmitter systems impacted by concurrent methamphetamine-fentanyl use

The addictive properties of concurrent methamphetamine and fentanyl use arise from separate, but overlapping, neurotransmitter systems. Methamphetamine directly elevates extracellular levels of all monoamines (Borowsky et al., 2001; Miller, 2011; Sulzer et al., 2005), while fentanyl activates MORs leading to elevated dopaminergic activation (Ford et al., 2006; Peng and Sandler, 1999). Despite these differences, research suggests that the MOR system likely underlies the synergistic effects of concurrent methamphetamine-fentanyl use. For instance, dopamine release in the NAc is modulated, in part, by VTA GABAergic neurons expressing MORs (Albanese and Minciacchi, 1983; Ford et al., 2006) and MORs play a critical role in modulating the development of behavioral sensitization to methamphetamine (Boutrel, 2008; Tien and Ho, 2011). Therefore, fentanyl agonism at these MORs may enhance the physiological and behavioral effects of methamphetamine and exacerbate its addiction vulnerability (Figure 1).

**Figure 1:**
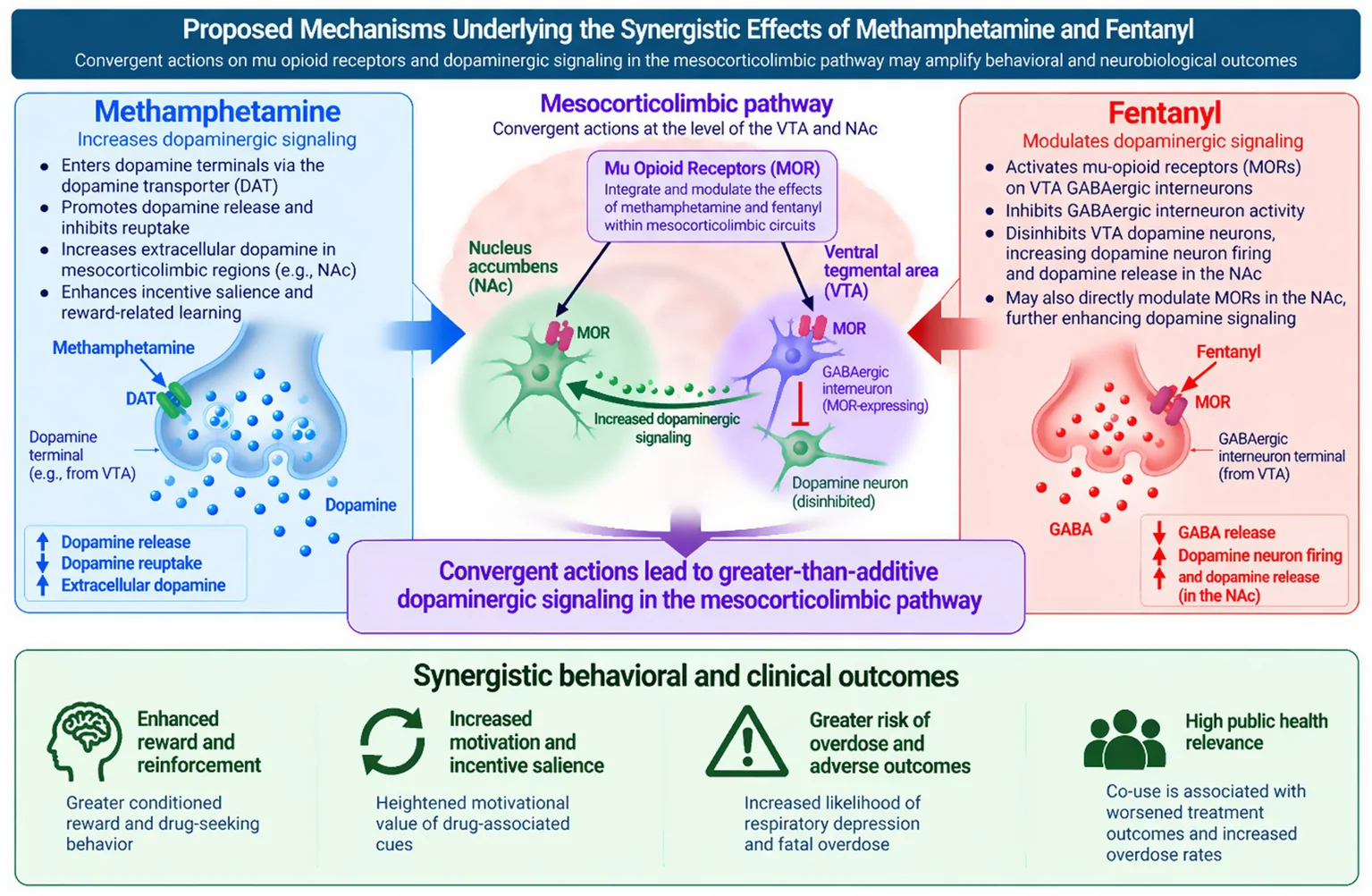
The mechanisms underlying the effects of methamphetamine (top left) and fentanyl (top right) on ventral tegmental area dopaminergic signaling are depicted, which may lead to synergistic activation of the mesolimbic pathway (top middle). Results stemming from the combined use of methamphetamine and fentanyl result in worsened addiction-related behavioral and clinical outcomes (bottom).

Rodent microdialysis studies have directly assessed how MOR agonism in the VTA influences mesolimbic dopaminergic activity. Microinjections of MOR agonists into the VTA increase concentrations of dopamine and dopamine metabolites throughout the ventral striatum, effects that are reversed by MOR antagonists (Devine et al., 1993). Furthermore, infusions of morphine into the VTA promote CPP, while infusions of KOR agonists promote conditioned place aversion (Phillips and LePiane, 1980; Bals-Kubik et al., 1993). Even when administered systemically, MOR agonists including fentanyl stimulate dopamine release in the NAc, effects that are attenuated by KOR agonists (Di Chiara and Imperato, 1988). In addition to influencing the striatum, VTA MOR and KOR systems also modulate the basolateral amygdala. MOR agonist infusions into the VTA inhibit GABAergic transmission and disinhibit dopamine neurons that project to the basolateral amygdala (Ford et al., 2006). Since both the NAc and basolateral amygdala modulate methamphetamine-induced reward and associative learning, opioidergic modulation of these regions may influence the progression of behavioral responses to methamphetamine, such as incentive salience and craving (Hedges et al., 2018; Li et al., 2023; Rocha and Kalivas, 2010; Yorgason et al., 2020).

The synergistic effects of methamphetamine and fentanyl may involve long-term neuroadaptations to the MOR system. During morphine withdrawal, enhanced MOR activity is observed in VTA, which may indicate a compensatory response to increased levels of cAMP during morphine withdrawal (Meye et al., 2012). Moreover, there are increases in levels of MOR mRNA in the NAc during morphine withdrawal (Zhou et al., 2006), while withdrawal from methamphetamine increases MOR expression throughout the striatum. Interestingly, these effects occur concurrently with the emergence of withdrawal-induced anxiety-like symptoms (Georgiou et al., 2016). Studies examining the role of psychostimulants in modulating MOR levels indicate that cocaine-induced dopamine release increases MOR mRNA levels in the NAc, indicating that indirect interactions between psychostimulants and opioid receptors produce neuroadaptations in the NAc (Azaryan et al., 1996). These neuroadaptive elevations in MOR mRNA levels could provide additional binding sites for opioids such as fentanyl, which could enhance the drug’s effects after chronic use and withdrawal. However, it is import to note that while repeated exposure to opioids and psychostimulants produce these neuroadaptations in opioid receptor systems, their contribution to the effects of combined methamphetamine–fentanyl exposure remains uncertain.

Disruptions in dopamine signaling also occur following chronic methamphetamine and opioid use. In humans, prolonged exposure to opioids attenuates dopamine release in the striatum, effects that are correlated with heightened reward thresholds and anhedonia (Blum et al., 2015; Shi et al., 2021; Yeh et al., 2012). Reduced levels of dopamine, however, may produce a compensatory adaptation, resulting in upregulation of D_1_ and D_2_ receptors. Indeed, postmortem analyses of opioid users reveal elevated D_1_ and D_2_ receptor expression in the VTA, NAc and amygdala (Sadat-Shirazi et al., 2018). Since these regions are crucial to methamphetamine-induced behaviors, these long-term adaptations may exacerbate the progression of methamphetamine addiction. In studies looking at combined methamphetamine and fentanyl use in rats, ex vivo slice voltammetry in the NAc reveals significant decreases in dopamine release and reuptake following methamphetamine-fentanyl exposure (Dawes et al., 2024). Similarly, male rats demonstrate a worsened hypodopaminergic state following combined methamphetamine-fentanyl exposure, compared to rats that received saline or methamphetamine alone (Dawes et al., 2024). The apparent differences between studies reporting enhanced dopaminergic activity following opioid exposure and those reporting hypodopaminergic adaptations may reflect specific differences in the timing and conditions under which dopamine function was assessed. Acute MOR activation may disinhibit VTA dopamine neurons and increase dopamine release in the NAc, while repeated drug exposure may produce compensatory neuroadaptations that reduce dopaminergic function during subsequent drug-free or withdrawal states. Dawes et al. (2024) assessed NAc dopamine function approximately 22 h following the final self-administration session, when animals were no longer under the acute pharmacological effects of either drug. Thus, the reductions in dopamine release and reuptake observed following methamphetamine-fentanyl self-administration likely reflect adaptations resulting from repeated drug exposure rather than the acute dopaminergic response to the drug combination. These findings suggest that repeated methamphetamine-fentanyl exposure produces increases in mesolimbic dopamine signaling during intoxication while promoting a compensatory hypodopaminergic state during withdrawal. Such state-dependent changes may contribute to heightened reward during drug exposure and diminished reward state during withdrawal, potentially increasing motivation for subsequent drug use.

Collectively, the available literature identifies MOR-dependent regulation of mesolimbic dopamine signaling as a plausible neurobiological framework for understanding interactions between methamphetamine and fentanyl. Direct evidence currently indicates that concurrent methamphetamine-fentanyl exposure can alter NAc dopamine dynamics, whereas evidence implicating VTA MOR signaling is derived primarily from studies of opioid pharmacology and MOR modulation of methamphetamine-related behaviors. Future studies directly manipulating MOR signaling during combined methamphetamine-fentanyl exposure will be necessary to determine whether this pathway mediates the neurochemical and behavioral effects of this drug combination.

## 5 Behavioral consequences of concurrent methamphetamine-fentanyl use

### 5.1 Alterations in positive reinforcement and incentive salience induced by concurrent methamphetamine-fentanyl use

Clinical reports indicate that individuals who use psychostimulants and opioids together often describe the combination as producing effects that are qualitatively different from either drug alone. Individuals reporting concurrent methamphetamine- or cocaine-opioid use have described the drugs as “balancing” one another, with opioids reducing stimulant-associated anxiety or restlessness and psychostimulants counteracting opioid-associated sedation (Baker et al., 2021; Ellis et al., 2018; Leri et al., 2003; Palmer et al., 2020; Rhodes et al., 2007). Although these reports provide important information regarding motivations for polydrug use, variation in dose, timing, route of administration and drug history limits conclusions regarding the pharmacological interactions between methamphetamine and fentanyl specifically.

Preclinical studies directly examining methamphetamine and fentanyl provide stronger evidence that exposure to the two drugs can alter reward- and reinforcement-related behaviors. In a concurrent-access self-administration paradigm, rats given access to methamphetamine and fentanyl adjusted their responding according to the relative reinforcing value of the available drugs, demonstrating that the two reinforcers can function as behavioral substitutes under some conditions (Seaman et al., 2022). Dawes et al. (2024) further report that rats self-administering methamphetamine and fentanyl do not increase inter-infusion intervals as fentanyl doses increase to the same extent observed during fentanyl administration alone, resulting in greater overall drug intake. These findings suggest that concurrent fentanyl and methamphetamine exposure can alter behavioral sensitivity to changes in fentanyl dose, although the mechanism responsible for this effect remains unclear.

Direct evidence of enhanced conditioned reward has also emerged from CPP studies. Paz-Ramos et al. (2024) found that doses of methamphetamine and fentanyl that are insufficient to establish CPP when administered individually produce significant place preference when administered together. This finding provides evidence that subthreshold doses of methamphetamine and fentanyl interact to produce a rewarding effect that is not observed following either drug alone. Importantly, these findings provide more direct support for enhanced reward following combined methamphetamine-fentanyl exposure than studies relying on other psychostimulant-opioid combinations.

Studies using methamphetamine with other opioids provide complementary evidence that stimulant-opioid combinations can alter reward and reinforcement. Combined methamphetamine and morphine administration produces CPP that is more resistant to extinction than place preference produced by either drug alone (Lan et al., 2009). Prior exposure to amphetamine or morphine can also enhance subsequent conditioned reward produced by the other drug (Lett, 1989), although such sequential exposure paradigms address effects of drug history rather than concurrent pharmacological interaction. Similarly, progressive-ratio self-administration studies indicate that methamphetamine-heroin combinations can maintain greater responding than methamphetamine alone (Ranaldi and Wise, 2000). These findings support the broader hypothesis that opioid and psychostimulant effects can interact behaviorally, while differences between morphine, heroin, fentanyl and individual psychostimulants limit direct generalization to methamphetamine-fentanyl co-use.

Broader psychostimulant-opioid literature provides additional mechanistic context. Cocaine-heroin combinations maintain higher levels of self-administration than cocaine alone, and opioid receptor antagonists reduce responding for psychostimulants under some experimental conditions. However, cocaine differs from methamphetamine in its pharmacological actions, and heroin and morphine differ from fentanyl in pharmacokinetics, receptor signaling and potency. Thus, these studies should therefore be viewed as supporting evidence for potential stimulant-opioid interactions rather than as direct demonstrations of methamphetamine-fentanyl effects.

The MOR system may underly some of these behavioral interactions. Naltrexone attenuates methamphetamine-induced CPP, cue-induced craving and methamphetamine seeking in both rodents and humans (Anggadiredja et al., 2004; Chiu et al., 2005; Ray et al., 2015; Roche et al., 2017; Wang et al., 2021). Additionally, MOR knockout mice exhibit reduced methamphetamine-induced behavioral sensitization (Shen et al., 2010). These findings demonstrate that opioid signaling can modulate methamphetamine-related reward and incentive processes. They do not, however, establish that fentanyl enhances these behaviors through the same mechanism. Rather, they identify MOR signaling as a candidate pathway that should be directly tested during combined methamphetamine-fentanyl exposure.

Collectively, the available evidence supports the conclusion that methamphetamine and fentanyl interact to alter reward- and reinforcement-related behavior. Direct studies demonstrate altered self-administration and enhanced conditioned reward during combined exposure, whereas studies involving other opioids and MOR manipulations provide supporting evidence for potential mechanisms. Additional experiments directly comparing individual and combined methamphetamine-fentanyl dose–response functions will be necessary to establish the conditions under which these effects are additive, synergistic or behaviorally independent.

### 5.2 Alterations in withdrawal symptoms and reinstatement of drug-seeking behaviors by concurrent methamphetamine-fentanyl use

Concurrent psychostimulant-opioid use is likely influenced by the aversive effects that emerge during intoxication and subsequent withdrawal. Qualitative studies indicate that some individuals use methamphetamine and opioids together because one drug is perceived to offset undesirable effects of the other. For example, individuals using methamphetamine and heroin have reported that methamphetamine can prolong desirable opioid effects or delay the subjective onset of withdrawal, while opioids may reduce stimulant-associated anxiety or agitation (Baker et al., 2021; Leri et al., 2003; Palmer et al., 2020; Silverstein et al., 2021). These reports suggest that relief from aversive internal states may contribute to continued polydrug use through negative reinforcement. However, because clinical patterns of use vary substantially and frequently involve additional substances, these findings cannot establish whether concurrent methamphetamine-fentanyl exposure reduces or exacerbates withdrawal symptoms.

Clinical studies further indicate that individuals with histories of both psychostimulant and opioid use often exhibit poorer psychiatric outcomes than individuals using psychostimulants alone, including greater levels of anxiety, depression, impulsivity, anhedonia, and psychotic symptoms (Burdzovic Andreas et al., 2015; Leri et al., 2003; Su et al., 2017; Willi et al., 2016). These findings demonstrate an association between polydrug exposure and greater psychiatric burden, but they should not necessarily be interpreted as evidence that withdrawal from concurrent methamphetamine-fentanyl use is more severe than withdrawal from either drug alone.

Direct preclinical evidence examining withdrawal following combined methamphetamine-fentanyl exposure remains scarce. Studies using other opioids provide preliminary evidence suggesting that simultaneous stimulant-opioid exposure may alter subsequent withdrawal. For example, rats exposed repeatedly to combined methamphetamine and morphine exhibit greater naloxone-precipitated jumping and escape behaviors than animals exposed to either drug individually (Kaka et al., 2014). These behaviors are consistent with increased somatic withdrawal severity, although they should not be interpreted exclusively as measures of anxiety. Because morphine differs pharmacologically from fentanyl, additional studies are needed to determine whether similar effects emerge following repeated methamphetamine-fentanyl exposure.

Prior opioid exposure and withdrawal may also alter subsequent drug reinforcement. Seaman and Collins (2021) reported that morphine withdrawal increased subsequent fentanyl self-administration relative to opioid-naïve or morphine-dependent conditions, whereas methamphetamine self-administration was not similarly increased. This sequential-exposure paradigm demonstrates that opioid history can modify subsequent fentanyl reinforcement but does not model concurrent methamphetamine-fentanyl exposure. Nevertheless, such findings emphasize that the behavioral effects of either drug may depend substantially on whether an individual is opioid-naïve, opioid-dependent or undergoing withdrawal.

Direct evidence from methamphetamine and fentanyl self-administration/extinction/reinstatement studies suggests that co-use may increase relapse rates following periods of abstinence. Rats previously trained to self-administer both drugs in a concurrent-access paradigm exhibit cross-reinstatement of extinguished drug seeking: fentanyl-associated cues or fentanyl priming injections reinstate methamphetamine seeking, whereas methamphetamine-associated cues or priming injections reinstate fentanyl seeking (Seaman et al., 2022). These findings indicate that experience with concurrent methamphetamine-fentanyl use may establish overlapping or interacting motivational associations such that exposure to one drug or its associated cues can promote seeking of the other drug.

Together, the available literature indicates that withdrawal state, prior drug history and drug-associated cues may all influence continued methamphetamine-fentanyl use. However, the evidence supporting these processes differs substantially across experimental paradigms. Cross-reinstatement following concurrent methamphetamine-fentanyl self-administration provides direct evidence of interacting drug-seeking processes, whereas studies of morphine withdrawal and broader psychostimulant-opioid use provide indirect evidence regarding the potential roles of dependence and negative reinforcement. Direct characterization of spontaneous and precipitated withdrawal following repeated methamphetamine-fentanyl exposure remains an important area for future investigation.

## 6 Discussion and future directions

Concurrent methamphetamine-fentanyl use represents a growing public health concern, yet the behavioral and neurobiological consequences of this specific drug combination remain comparatively understudied. The available literature nevertheless provides emerging evidence that methamphetamine and fentanyl interact in ways that alter reward, reinforcement, dopamine signaling and subsequent drug-seeking behavior. Importantly, however, the strength of this evidence varies considerably across experimental paradigms. Direct studies involving combined or concurrent methamphetamine-fentanyl exposure provide the strongest evidence regarding the interaction between these drugs, whereas studies examining methamphetamine with other opioids, prior exposure to one drug, or pharmacological and genetic manipulations of opioid systems provide complementary mechanistic information that should be interpreted more cautiously.

Direct preclinical studies indicate that concurrent methamphetamine-fentanyl exposure produces behavioral effects that differ from those observed following administration of either drug alone. Combined doses of methamphetamine and fentanyl that are insufficient to produce CPP individually establish significant place preference when administered together, providing evidence for enhanced conditioned reward under specific dose conditions (Paz-Ramos et al., 2024). Concurrent-access self-administration studies further demonstrate interactions between the reinforcing effects of methamphetamine and fentanyl. Rats provided access to both drugs allocate responding according to their relative reinforcing value and exhibit altered sensitivity to changes in fentanyl dose during combined exposure (Dawes et al., 2024; Seaman et al., 2022). Following extinction, experience with concurrent access also produces cross-reinstatement, such that fentanyl-associated stimuli promote methamphetamine seeking and methamphetamine-associated stimuli promote fentanyl seeking (Seaman et al., 2022). Together, these findings demonstrate that methamphetamine and fentanyl interact at multiple stages of addiction-related behavior, although the magnitude and direction of these interactions likely depend on dose, schedule of drug availability, exposure history and the behavioral paradigm.

Emerging neurobiological evidence similarly indicates that combined methamphetamine-fentanyl exposure can alter mesolimbic dopamine function. Concurrent self-administration produces changes in dopamine release and reuptake within the NAc, including a more pronounced hypodopaminergic phenotype relative to methamphetamine exposure alone (Dawes et al., 2024). The neural mechanisms responsible for these effects have not yet been established. Methamphetamine directly increases extracellular monoamine concentrations through actions involving TAAR1, VMAT2 and monoamine transporters, whereas fentanyl activates MORs that can regulate dopamine transmission through inhibitory circuitry within the VTA. Because MOR activation on VTA GABAergic neurons can disinhibit dopamine neurons and systemic MOR agonists can alter NAc dopamine concentrations, MOR-dependent modulation of mesolimbic dopamine represents one plausible mechanism through which fentanyl could influence the effects of methamphetamine. However, direct manipulation of this pathway during concurrent methamphetamine-fentanyl exposure will be required before a causal role for VTA MOR signaling can be established.

Evidence obtained using other opioids further supports the possibility that opioid signaling modifies methamphetamine-related behaviors. Morphine and heroin enhance or alter methamphetamine reward and reinforcement in several preclinical paradigms, while MOR antagonism and genetic deletion attenuate methamphetamine-induced CPP, cue-associated responding and behavioral sensitization (Anggadiredja et al., 2004; Chiu et al., 2005; Lan et al., 2009; Ranaldi and Wise, 2000; Shen et al., 2010; Wang et al., 2021). Similarly, human studies indicate that naltrexone reduces cue-induced craving and subjective responses for methamphetamine (Ray et al., 2015; Roche et al., 2017). These findings indicate that opioid systems contribute to several methamphetamine-related behavioral processes and provide mechanistic support for investigating MOR signaling during methamphetamine-fentanyl exposure. Nevertheless, differences among fentanyl, morphine and heroin, as well as between methamphetamine and cocaine, limit the extent to which these findings can be generalized directly to concurrent methamphetamine-fentanyl use.

Clinical studies suggest that motivations for psychostimulant-opioid co-use may extend beyond enhancement of positive subjective effects. Individuals using methamphetamine and opioids together frequently report that one drug can offset undesirable effects of the other, including stimulant-associated anxiety or agitation and opioid-associated sedation or withdrawal-related discomfort (Baker et al., 2021; Ellis et al., 2018; Leri et al., 2003; Palmer et al., 2020; Silverstein et al., 2021). Such patterns raise the possibility that both positive and negative reinforcement contribute to continued co-use. However, clinical polydrug use is heterogeneous, and the dose, temporal relationship and history of exposure to each drug are often difficult to establish. Consequently, these reports are informative regarding motivations for co-use but do not establish the pharmacological consequences of simultaneous methamphetamine-fentanyl exposure.

Based on the current literature, several important questions need to be addressed. First, systematic dose–response studies are needed to determine the conditions under which methamphetamine-fentanyl combinations produce additive, synergistic or independent effects on reward and reinforcement. Studies should directly compare individual and combined doses across CPP, fixed- and progressive-ratio self-administration and drug-choice procedures. Such approaches will help determine whether enhanced responding reflects increased reinforcing efficacy, changes in drug discrimination or sensitivity, altered satiety or other behavioral processes.

Second, future work should characterize how the temporal relationship between methamphetamine and fentanyl exposure influences behavioral outcomes. Simultaneous administration, sequential administration within a session, concurrent availability as separate reinforcers and a prior history of exposure to one drug represent biologically distinct conditions. Direct comparison of these paradigms would clarify whether the effects of co-use depend on acute pharmacological interaction or on neuroadaptations produced by previous drug exposure. Particular attention should be given to opioid-naïve, and opioid-dependent and withdrawn states, because opioid history can substantially modify subsequent fentanyl reinforcement and may alter responses to methamphetamine-fentanyl combinations.

Third, withdrawal following repeated combined methamphetamine-fentanyl exposure remains poorly characterized. Existing evidence from other psychostimulant-opioid combinations suggests that co-exposure may alter the expression of somatic and affective withdrawal signs, but direct studies with methamphetamine and fentanyl are needed. Future experiments should distinguish spontaneous from precipitated withdrawal and separately assess somatic withdrawal, anxiety-like behavior, anhedonia, motivational deficits and changes in natural reward processing. Longitudinal studies could further determine how these outcomes evolve across acute withdrawal and prolonged abstinence, and whether they predict subsequent reinstatement or escalation of drug taking.

Finally, mechanistic studies should proceed in parallel with behavioral characterization of the drug combination. MOR signaling within the VTA and its downstream targets represents one particularly strong candidate pathway, but this hypothesis should be tested directly rather than inferred from downstream dopamine measurements or studies involving other opioids. Region-specific pharmacological manipulations, receptor-selective genetic approaches, electrophysiology, fiber photometry and direct measurements of dopamine release could help distinguish changes in VTA neuronal activity from alterations in terminal dopamine release within regions such as the NAc. Lastly, studies should also examine whether repeated methamphetamine-fentanyl exposure produces neuroadaptations that differ from those induced by either drug individually and whether these adaptations vary according to dependence or withdrawal state.

Collectively, the available evidence indicates that concurrent methamphetamine-fentanyl exposure can alter reward, reinforcement, dopamine signaling and drug-seeking behavior, but the mechanisms responsible for these effects remain incompletely characterized. The existing literature supports MOR-dependent modulation of mesolimbic dopamine signaling as a plausible framework for understanding these interactions rather than an established mechanism unique to concurrent methamphetamine-fentanyl use. Clearly distinguishing direct evidence from findings obtained using other opioids, sequential exposure and mechanistic manipulations will be essential as this literature develops. Continued investigation of methamphetamine-fentanyl co-use across behavioral, physiological and neurobiological levels will help determine when these drugs interact synergistically and identify neural mechanisms that may contribute to the heightened addiction vulnerability associated with their combined use.

## Glossary

MOR: μ opioid receptor
CNS: Central nervous system
CPP: Conditioned place preference
CRF: Corticotropin-releasing factor
cAMP: Cyclic adenosine monophosphate
DAMGO: [D-Ala2, N-Me-Phe4-Gly5-ol]-enkephalin
DAT: Dopamine transporters
EPM: Elevated plus-maze
HPA axis: Hypothalamic-pituitary-adrenal axis
MRI: Magnetic resonance imaging
NET: Norepinephrine transporters
NAc: Nucleus accumbens
PPTg: Pedunculopontine tegmental nucleus
PKA: Protein kinase A
PKC: Protein kinase C
PET: Positron emission tomography
SERT: Serotonin transporters
TAAR1: Trace amine associated receptor 1
VTA: Ventral tegmental area
VMAT2: Vesicular monoamine transporter 2
